# *Bartonella quintana* and *Coxiella burnetii* as Causes of Endocarditis, India

**DOI:** 10.3201/eid1407.071374

**Published:** 2008-07

**Authors:** Nandhakumar Balakrishnan, Thangam Menon, Pierre-Edouard Fournier, Didier Raoult

**Affiliations:** * University of Madras, Taramani, Chennai, India; † Universite de la Mediterranee, Marseille, France

**Keywords:** blood culture negative endocarditis, endocarditis, Bartonella, Coxiella burnetii, serology, India, letter

**To the Editor:** In industrialized countries, blood culture is negative for 2.5%–31% of infectious endocarditis cases (*1*). In developing countries such as South Africa (*2*), Algeria (*3*), and Pakistan (*4*), culture is negative for 48% to 56%. Negative cultures delay diagnosis and treatment, which profoundly affects clinical outcome. Negative blood cultures commonly result from previous administration of antimicrobial drugs, right-sided endocarditis, or fastidious or noncultivable pathogens (*1*). Our aim was to identify fastidious agents of blood culture–negative endocarditis by serology**.** Because of recent attention to zoonotic microorganisms as agents of this condition in developing countries (*1*), we investigated the prevalence of *Coxiella burnetii*, *Bartonella* spp., and *Brucella melitensis* among endocarditis patients in India.

We cultured blood from 111 patients admitted to the Government General Hospital, Chennai, India, from August 2005 through December 2006, with a diagnosis of infectious endocarditis according to the Duke criteria (*5*). Informed consent was obtained from all patients. Three blood samples from each patient, collected at hourly intervals, were inoculated into brain–heart infusion broth supplemented with 0.04% sodium polyanethol sulfonate (HiMedia, Mumbai, India). Cultures were incubated at 37°C in a 5% CO_2_ atmosphere for 14 days and checked each day for turbidity. Subcultures were made on 5% sheep blood and MacConkey agar at 37°C at 24 hours, 48 hours, and when culture broth appeared turbid.

Blood cultures were negative for 80 (72%) of the 111 patients. Serum from 63 patients was available for serologic testing. Of these patients, 30 were male and 33 were female; age range was 5–65 years and mean age was 25.5 years. Endocarditis involved the native valve for 60 (95.23%) and a prosthetic valve for 3 (4.76%). The most frequent predisposing factor was rheumatic heart disease, found in 38 (60.31%). Of the 60 with native valve endocarditis, the involved valve was mitral for most (36, 60.0%), followed by aortic (8, 13.33%), tricuspid (7, 11.66%), and pulmonary (1, 1.66%); 8 (13.33%) had both valvular and nonvalvular endocarditis. Of the 3 patients with prosthetic valve endocarditis, the involved valve was mitral for 2 and aortic for 1.

Serum samples were screened for *Bartonella* spp. and *C. burnetii* by microimmunofluorescence (*6*,*7*). A diagnosis of endocarditis was based on an immunoglobulin (Ig) G titer >800 to phase I *C. burnetii*; this titer has a positive predictive value of 98% (*6*). A diagnosis of *Bartonella* infection was based on the combination of a positive microimmunofluorescence titer (IgG to *B. quintana* or *B. henselae* >200) and a Western blot profile consistent with endocarditis (*8*).

Identification of the causative species was obtained by Western blot after cross-adsorption with either *B. henselae* or *B. quintana* antigens (*8*). Antibodies to *B. melitensis* were detected by agglutination by using the Rose Bengal and *Brucella* Wright tests (both from BioRad, Hercules, CA, USA). Of the 63 patients, 9 had a positive antibody response against a tested antigen (Table): 1 to phase I *C. burnetii* and 8 to *Bartonella* spp. (IgG >200). Of these, 7 had a 1-fold dilution higher titer to *B. quintana* than to *B. henselae*, including 1 with a low-level cross-reaction with *C. burnetii*, and 1 had identical titers to both. For all 8 patients, Western blot results were consistent with *Bartonella* endocarditis. For 7, cross-adsorption identified *B. quintana* as the causative species; for the other, the infecting *Bartonella* species remained undetermined because adsorption with *B. quintana* and *B. henselae* antigens removed all antibodies. Serologic results for *B. melitensis* were negative for all patients.

**Table:** Clinical findings and causative agent for 9 patients with blood culture–negative endocarditis, India, August 2005–December 2006*

Patient age, y/sex	Underlying cardiac condition	Echocardiographic findings	IgG titer to *Bartonella* spp.	IgG titer to *Coxiella burnetii* phase I	Causative agent
25/F	Right atrium fistula	Vegetation attached to tricuspid valve	400	100	*Bartonella quintana*
46/M	Rheumatic heart disease	Vegetation attached to anterior mitral leaflet	0	800	*Coxiella burnetii*
14/M	Rheumatic heart disease	Vegetation attached to tip of anterior mitral leaflet	200	0	*B. quintana*
13/M	Rheumatic heart disease	Vegetation attached to anterior mitral leaflet	200	0	*B. quintana*
28/M	Bicuspid aortic valve disease	Vegetation attached to anterior coronary cusp of aortic valve	400	0	*B. quintana*
30/M	Rheumatic heart disease	Vegetation attached to both anterior and posterior mitral leaflet extending to chordae tendinae	200	0	*B. quintana*
50/F	Rheumatic heart disease	Vegetation attached to noncoronary cusp of aortic valve	400	0	*Bartonella* spp.
40/M	Bicuspid aortic valve disease	Calcified aortic valve	400	0	*B. quintana*
40/M	Double chamber right ventricle and subaortic perimembranous ventricular septal defect	Vegetation attached to right atrium anterior leaf of tricuspid valve and lateral cusp of pulmonary valve	800	0	*B. quintana*

*Ig, immunoglobulin.

*B. quintana* is mostly associated with human body lice but has also been found in fleas (*9*). The predisposing factors for *B. quintana* endocarditis are homelessness, alcoholism, and exposure to body lice (*10*). For our patients, the common predisposing factors were poor hygiene and low socioeconomic status, which may expose them to ectoparasites including lice and fleas. In contrast with previous study findings, *B. quintana* infectious endocarditis developed on preexisting valvular lesions in all patients (*10*). This finding may reflect a different clinical evolution than in Europe, where studies have suggested that *B. quintana* infectious endocarditis followed chronic bacteremia in patients who did not have previous valvular defects (*10*).

In summary, prevalence of negative blood culture among patients with infectious endocarditis was high (72%). The most commonly associated risk factor was rheumatic heart disease (Table). *C. burnetii* and *Bartonella* spp. were responsible for 8% of all infectious endocarditis cases and 14% of blood culture–negative cases. No case of infectious endocarditis caused by *B. melitensis* was identified.

Our preliminary study suggests that zoonotic agents, especially *Bartonella* spp., are prevalent causative organisms of blood culture–negative endocarditis in India. We recommend serologic screening for antibodies to zoonotic microorganisms as diagnostic tools for this disease in India.
